# Structuring the skies: Diel dynamics of migratory animal movement in the lower atmosphere

**DOI:** 10.1002/ecy.70247

**Published:** 2025-11-18

**Authors:** Silvia Giuntini, Carolyn S. Burt, Annika L. Abbott, Carrie Ann Adams, Maria Carolina T. D. Belotti, Yuting Deng, Miguel F. Jimenez, Jeffrey F. Kelly, Subhransu Maji, Meredith Nash‐Martin, Sam Simon, Daniel Sheldon, Kyle G. Horton

**Affiliations:** ^1^ Environment Analysis and Management Unit ‐ Guido Tosi Research Group, Department of Theoretical and Applied Sciences University of Insubria Varese Italy; ^2^ Department of Forestry and Natural Resources Purdue University West Lafayette Indiana USA; ^3^ Department of Natural Resources and Environmental Sciences University of Illinois Urbana‐Champaign Urbana Illinois USA; ^4^ Department of Biology Carleton University Ottawa Ontario Canada; ^5^ Natural Resource Ecology and Management Department Oklahoma State University Stillwater Oklahoma USA; ^6^ Cornell Lab of Ornithology Ithaca New York USA; ^7^ Department of Conservation and Science Lincoln Park Zoo, Urban Wildlife Institute Chicago Illinois USA; ^8^ School of Biological Sciences, University of Oklahoma Norman Oklahoma USA; ^9^ Manning College of Information and Computer Sciences, University of Massachusetts Amherst Amherst Massachusetts USA; ^10^ Department of Fish, Wildlife, and Conservation Biology Colorado State University Fort Collins Colorado USA

**Keywords:** aeroecology, airspace, avian migration, niche space, radar remote sensing

## Abstract

Earth's lower atmosphere is a vital ecological habitat, home to trillions of organisms that live, forage, and migrate through this medium. Despite its importance, this space is seldom considered a primary habitat for ecological or conservation prioritization, making it one of the least studied environments. However, it plays a crucial role as a global conduit for the transfer of biomass, weather, and inorganic materials. Fundamental research is essential to address core ecological questions related to the ecological consequences of this habitat's intricate spatial and temporal structure. To advance our understanding of airspace use by migratory animals, we analyzed over 108 million 5‐min radar observations from 143 NEXRAD sites, focusing on 24‐h diel cycles across the contiguous United States. This extensive dataset, spanning from 1995 to 2022, allowed us to quantify aerial space use by systematically identifying peak activity times, the portion of the airspace that contained the majority of migration activity, and the percentage of migrants passing across diurnal and nocturnal diel cycles. We found that airspace is used predominantly during nocturnal periods in both spring and autumn (88%), while summer exhibited a more balanced distribution (54% nocturnal). Additionally, the percentage of nocturnal activity increased with latitude in spring and autumn but decreased in summer. Peak aerial activity typically occurred about 4 h after local sunset in both spring and autumn, with variations based on latitude and longitude. During these peak times, on average, half of the aerial movement was confined within a vertical band of 516 meters, starting around 355 m above ground level. Our research underscores the need to view the lower atmosphere as a structured habitat with significant ecological importance.

## INTRODUCTION

Airspaces are a woefully understudied global habitat that have wide‐ranging ecological implications. For more than a century, characterizing habitat use and assessing habitat suitability has been an ecological priority across a rich diversity of taxa and environments (Wiens et al., 2009), however with one primary omission—airspaces. The ocean is commonly regarded as the largest habitat on Earth (Levin et al., 2019; Warrant, 2004; Witman et al., 2008), covering approximately 71% of Earth's surface (Charette & Smith, 2010). However, in terms of volume, the troposphere (i.e., the first 10–12 km in altitude above ground level) vastly surpasses the ocean, with an estimated 6.6 billion cubic kilometers (Fabian & Dameris, 2014) compared to the ocean's 1.34 billion cubic kilometers (Costello et al., 2010). As a result, airspace is the largest global habitat, and while our perception of this space often does not fit our classic notion of a habitat, it fits all the characteristics of an ecological habitat (Diehl, 2013). Consequently, airspaces represent a crucial environment for aerial species, including, but not limited to, birds, bats, and insects. Migration, foraging, and even sleep are some of the fundamental ecological behaviors occurring in airspaces (Dokter et al., 2013; Dreelin et al., 2018; Horton et al., 2016). Flight altitudes can reach up to 8 km above Earth's surface, as demonstrated by species such as the Great Snipe (*Gallinago media*), which achieves such elevations even in the absence of orographic constraints (Lindström et al., 2021). For thousands of migratory species of birds, insects, and bats, airspaces are a primary habitat (Hu et al., 2016; Van Doren & Horton, 2018; Werber et al., 2023)—their fitness is directly dependent on the resources that airspaces provide (e.g., wind profit, thermal updrafts, food).

Radars have long been essential tools for studying the spatiotemporal distribution of birds, bats, and insects in flight (Belotti et al., 2023; Gauthreaux, 1970; Horton et al., 2023; Stepanian et al., 2020; Stepanian & Wainwright, 2018). They have been used to investigate the influence of wind on bird migration speed (Nussbaumer et al., 2022), shifts in bat phenology (Stepanian & Wainwright, 2018), and diverse insect migration behaviors, including dawn departures and wind‐assisted transport (Chapman et al., 2008; Chapman, Klaassen, et al., 2011). These radars offer unprecedented insight into the aerial movements of flying animals, enabling the study of their migratory behaviors with exceptional spatiotemporal precision—and thus, offer a way to characterize the habitat use by migratory organisms across space and time. Numerous questions persist regarding the temporal distribution of aerial migrations and preferred flight altitudes. Variation in the use of airspaces during different times of the day—diurnal and nocturnal—can have a significant impact on migration dynamics, habitat preferences, and abiotic conditions. While most bird species migrate at night (Alerstam, 2009; Horton, Nilsson, et al., 2019; Libby, 1899), the daily timing of migration is subject to influences from various environmental and hormonal factors, with similar patterns observed in insects (Eikenaar & Schmaljohann, 2015; Hakbong et al., 2021). Moreover, migratory flight altitude exhibits considerable variability, being influenced by a multitude of biotic and abiotic factors (Kemp et al., 2023; Knop et al., 2023; Sjöberg et al., 2023). It follows naturally to assume that aerial habitat use varies spatially and temporally across the continental United States throughout the 24‐h cycle.

Airspaces are becoming increasingly populated with structures such as buildings, communication towers, and wind turbines (Loss et al., 2013; Nilsson et al., 2021; Scott et al., 2023). Beyond these anthropogenic structures, airspaces are increasingly polluted with smoke and debris from wildfires (Buchholz et al., 2022), gaseous pollutants, and light pollution (Burt, Kelly, Trankina, et al., 2023; Horton, Nilsson, et al., 2019). Light pollution can serve as an attractant for migratory birds, drawing birds into large concentrations (Van Doren et al., 2017), and can exacerbate fatal collisions with structures, which approaches a billion birds annually in North America (Loss et al., 2013; Van Doren et al., 2021). Understanding airspace use during active migration is critical for reducing collision risk and mitigating anthropogenic pollutants. Therefore, it is necessary to gain insight into the organization of these complex habitats and to define their use, especially considering that agencies are increasingly required to address emerging conservation challenges in State Wildlife Action Plans (SWAPs), yet often lack sufficient data to guide effective policy and planning (Fontaine, 2011).

Here, we describe the aerial habitat use of migratory taxa during spring, summer, and autumn across 28 years. We analyzed US weather surveillance radar data to quantify the distribution of aerial activity of migratory aerofauna (i.e., birds, bats, and insects) at a continental scale. While recent years have seen progress in analyzing ecological dynamics in the aerial environment (La Sorte et al., 2015; Péron et al., 2020; Roemer et al., 2019), broad‐scale studies specifically examining active migration remain largely unexplored, particularly across 24‐h cycles. We predict that airspace is predominantly used at night by migratory organisms and that aerial migratory activity varies with latitude, with a higher percentage of nocturnal activity at northern latitudes due to the greater concentration of insects—typically active during the day—at lower latitudes (Tielens & Kelly, 2024). We aim to leverage the archive of radar remote sensing data to characterize the use of the aerial habitat across time and space.

## METHODS

### Weather surveillance radar processing

To quantify aerial habitat use, we processed weather surveillance data from 143 NEXRAD sites from 1995 to 2022 (28 years) across the continental United States. For all radars, we downloaded and processed radar scans during all hours of the day to understand differences in diurnal and nocturnal activity patterns, defining the nocturnal phase as the time at which the sun was below the horizon (i.e., sun angle was < 0). We used the R suntools package (Bivand & Luque, 2023) to calculate sunset times based on the latitude and longitude of the radar site. We defined spring from March 1 to June 15 (106 days), summer from June 16 to July 31 (45 days), and autumn from August 1 to November 15 (106 days). This definition of the two migratory seasons, spring and autumn, was chosen to broadly encompass almost all migratory movements—day or night—of aerial animals in the United States, particularly birds. The nonmigratory period, summer, was included as a benchmark to compare aerial habitat use during and outside migration.

For every radar scan, we generated vertical profiles of reflectivity. Prior to assembling these profiles, we removed precipitation using the MISTNET algorithm (Lin et al., 2019), a convolutional neural network that uses radar reflectivity factor, radial velocity, and spectrum width as input features to classify scattering volumes (e.g., precipitation, biology). Once contamination of plan position indicator (PPI) layers was removed from radar reflectivity factor and radial velocity, we assembled the five lowest elevation scans from 2.5 to 50 km range to build profiles up to 3000 m above ground level at 100‐m intervals. While the radars do miss some proportion of the airspace at the lowest reaches, the lowest elevation scans (~0.4–0.6°) do collect a substantial number of viable voxels in the 0‐ to 100‐m vertical profile catchment. We determined that at the lowest interval (0–100), 25,950 + 5872 (SD) voxels were used, on average, to estimate reflectivity and construct a velocity azimuth display (i.e., flight direction and ground speed). Across all 30 height intervals, the mean number of voxels available for estimation was 20,089 (https://doi.org/10.6084/m9.figshare.29688863.v1). We then calculated reflectivity traffic rate, computed by vertically integrating reflectivity multiplied by groundspeed (Horton, Van Doren, et al., 2019). This metric represents the total radar cross‐section in square centimeters crossing over a 1‐km transect in 1 h, where the transect is adaptively chosen for each height bin to be perpendicular to the mean direction of travel in that height bin.

Because radar scans are not collected at regular temporal intervals (e.g., ~every 4–10 min), we regularized the temporal sampling to every 5 min using linear interpolation. Specifically, we interpolated measures to the nearest measurement if the temporal gap was less than 1 h; otherwise, the measure was left empty. This step was important to ensure total activity indices could be compared across dimensions of interest, including across diel periods, across seasons, or across stations. We removed 5‐min samples if more than 25% of the scattering volumes were classified as rain and only included sampling days, defined as scans between 20:00 UTC and 19:59 UTC the following day, if at least 90% of 5‐min scans remained after filtering.

### Calculating percentages of diurnal versus nocturnal activity

We calculated the sum of passage during diurnal and nocturnal phases for all radar–season–year combinations. We calculated the percent activity in each phase at the seasonal level, rather than the percent of activity during diurnal and nocturnal phases per sampling day. This was done to ensure that summaries reflected the dominant behavior of the migration system, rather than the mean behavior per sampling day, which would give even weight to days. For instance, if 10 birds (9 diurnal, 1 nocturnal, 10% nocturnal activity) were flying on one day and 10,000 birds (1000 diurnal, 9000 nocturnal, e.g., 90% nocturnal activity) on another, summarizing by daily phases would mask the differences in intensity of aerial activity. This would result in a daily summary metric showing 50% nocturnal activity versus 89.9% across the season.

### Calculating the temporal window of peak activity

To determine when aerial activity peaked throughout the 24‐h period, we fitted generalized additive models (GAMs) to time series of traffic rates, with time after sunset as the predictor variable. We fitted models to each radar–season–year combination and only included days that were in the top quartile of mean activity to ensure that timeseries represented periods of strong aerial activity. From model predictions, we determined the hour after sunset where activity peaked; this yielded a time for each radar–season–year (e.g., 4.1 h after sunset for KTLX in spring 2022). We only maintained predictions if variance explained was greater than 20% and the model was fit on at least three sampling days. From radar–season–year estimates, we calculated the SD of peak time in hours.

### Calculating the spatial envelope of peak activity

To characterize the use of airspace, including space, we also calculated flight heights. However, rather than simply determining flight heights where traffic rates peaked, we sought to determine the range of heights that encompassed 50% of migrant passage during envelopes for peak activity. Choosing the 50% range was intended to focus specifically on the envelope that captured the majority of migratory activity (e.g., Horton et al., 2021), thus identifying the spatial peak of migration. Additionally, we also provide 75% envelopes in Horton, 2025c but focus our results on 50% envelopes herein. For each radar–season–year combination, we extracted the same sampling days used to calculate the peak activity period (e.g., top quartile of mean activity). For these sampling days, we further restricted our examination to the predicted time of peak activity ± 1 SD. For each of these radar scans, we determined the minimum vertical space that captured 50% of the aerial activity. We summarized these ranges by taking the mean of the upper and lower heights. Additionally, we also provide the 50% envelopes in Horton, 2025c that captured 50% of summed traffic rates for all scans in the peak activity period, rather than the means of many scan‐level envelopes.

## RESULTS

**FIGURE 1:**
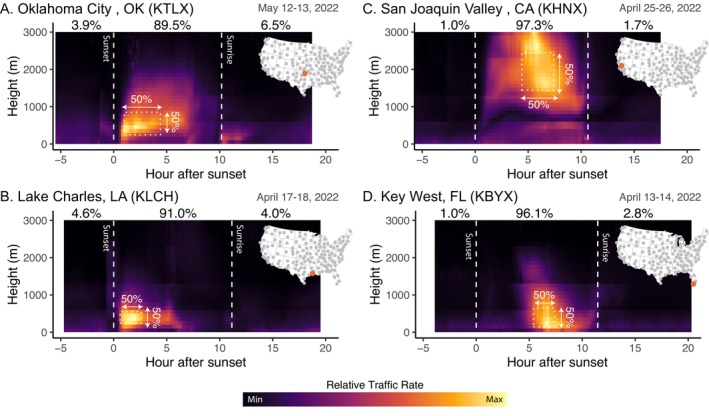
Vertical profiles of the square root of migration activity across 24‐h periods for four NEXRAD stations: (A) Oklahoma City, OK (KTLX), (B) Lake Charles, LA (KLCH), (C) San Joaquin Valley, CA (KHNX), and (D) Key West, FL (KBYX). For each 24‐h period, we reference observations relative to local sunset, but also delineate local sunset and sunrise by a dotted line. Sunset and sunrise are defined by a solar angle of 0°. For these four observation periods, we identified both the temporal (hour after sunset) and spatial (height above ground level) range that captured 50% of the summed traffic rate and the height interval that captured 50% of activity. Inset maps show the location of the NEXRAD installation.

**FIGURE 2:**
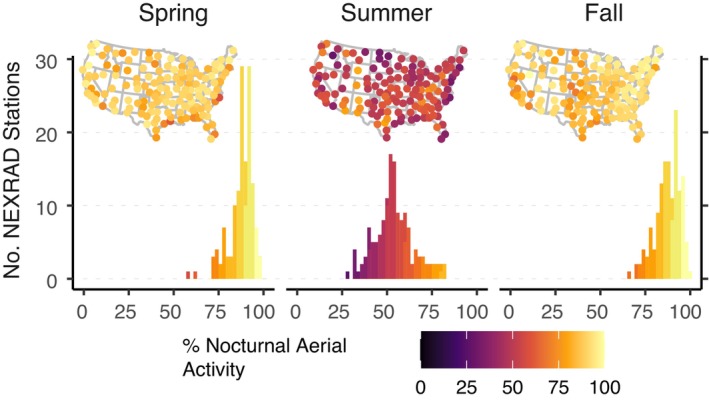
Percent of cumulative migration passage across spring (March 1–June 15), summer (June 16–July 31), and autumn (August 1–November 15) for nocturnal diel periods. We present both the frequency tallies and a map of locations shaded by percent of aerial activity by diel phase for each season. The nocturnal phase is defined by a solar angle <0°.

In total, we analyzed 108,440,281 five‐minute weather surveillance radar scans to characterize the airspace use of aerial migrants across the continental United States. On average, each station recorded 26 years of temporal coverage, ranging between 12 and 28 years, with an average of 29,111 samples per station per year. During spring and autumn, every station showed more cumulative activity during the nocturnal phase (e.g., Figures 1 and 2), with 87.7% of spring and 87.6% of autumn activity occurring during nocturnal phases. However, summer showed a more even balance across diurnal and nocturnal phases, with 70% of stations showing more activity during nocturnal phases (100 stations) and 54% of activity occurring during the nocturnal phase. A greater percentage of nocturnal activity was found with increasing station latitude in spring (slope = 0.257, *p* = 0.001) and autumn (slope = 0.250, *p* = 0.016), but a decreasing percentage with increasing latitude in summer (slope = −0.423, *p* = 0.011). Mean traffic rates were 1.3 times higher in autumn as compared to spring, and 6.3 times higher compared to summer, while spring mean traffic rates were 4.6 times greater than summer.

**FIGURE 3:**
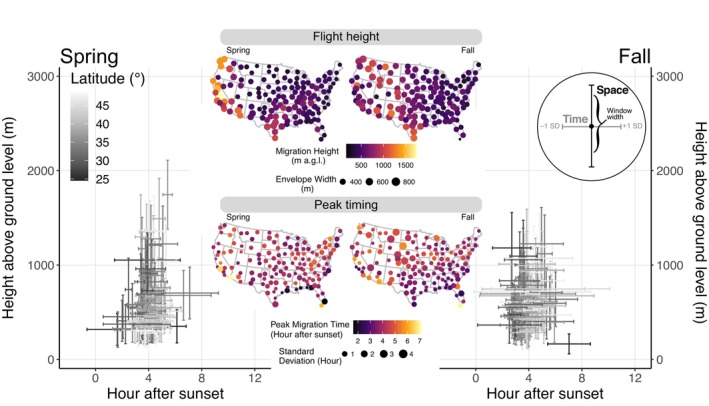
Spring and autumn spatiotemporal airspace use measured at 143 NEXRAD installations from 1995 to 2022. Spring and autumn biplots show temporal windows during which migration peaked ±1 SD and mean height envelopes that captured 50% of migration traffic rates during peak temporal intervals. Migration height maps show NEXRAD locations shaded by height above ground level and point size scaled to flight height envelope width. Peak migration time maps show NEXRAD locations shaded by the hour after sunset when migration traffic rates peaked, and size scaled to the SD of the temporal peak estimate.

Aerial activity peaked approximately 4.1 ± 0.7 (±SD) hours after local sunset in spring and 4.0 ± 0.8 (±SD) hours after local sunset in autumn, and we found no significant difference across these two seasons, both when examining overall seasonal differences (*t*_284_ = 0.622, *p* = 0.534) and examining paired differences (*t*_142_ = 0.761, *p* = 0.448) (Figure 3). Peak timing was earlier at more northerly latitudes in the spring (slope = 0.026, *p* = 0.025) and showed no longitudinal pattern (slope = −0.005, *p* = 0.200); in autumn, peak timing was later at more northerly latitudes (slope = 0.0412, *p* < 0.001) and later at more western longitudes (slope = −0.016, *p* < 0.001). During the summer, peak timing was earlier and more variable, but also peaked during the nocturnal phase, 3.6 ± 2.5 (±SD) hours after local sunset.

During periods of peak aerial activity, 50% of aerial activity occurred within a mean vertical envelope of width 516 ± 264 m (±SD), with a mean starting height of 355 ± 440 (±SD) m above ground level (Figure 3). During both spring and autumn, longitude was a consistent predictor of envelope width and the starting height of the envelope, with sites further west showing higher flight heights and wider envelopes of airspace that encompassed 50% of aerial activity. Due to the lack of concentration in the temporal phase and a nearly threefold increase in variability, we chose not to characterize flight heights during the summer season because we did not think they would be reliable.

## DISCUSSION

Quantifying the use of the aerial environment is a crucial step toward understanding how airspaces function as critical ecological habitats. Winged migration is dependent on this habitat—our study demonstrates how these spaces are structured, both in time and space. Throughout North America, most migratory bird species, all bats, and many insect species are characterized as nocturnal migrants. For instance, approximately 80% of migratory bird species migrate under the cover of darkness (Horton, Nilsson, et al., 2019). Our analysis further reinforces this point by using weather surveillance radar data to quantify aerial activity amassed from over 100 million remote sensing observations, showing that 88% of aerial activity between 0 and 3000 m above ground level occurred during the nocturnal diel phase.

Although our radar measures encompass all aerial biomass, that is, birds, bats, and insects, it is important to specify that most of the recorded activity can likely be attributed to migrating birds and insects and, to a lesser extent, bats, with some exceptions to specific airspaces (e.g., south‐central Texas). On a continental scale, the number of bats in flight is likely negligible compared to the billions of birds and trillions of insects detected (Dokter et al., 2018; Stepanian & Wainwright, 2018; Tielens & Kelly, 2024). Using portable radars at 10 sites throughout Israel, Werber et al. (2023) showed that bats made up an average of 14.6% of aerial detections; however, when accounting for migration traffic rates, bat traffic rates were nearly an order of magnitude less than birds and insects. Additionally, our approach of examining migration traffic rates likely excludes a portion of non‐migratory birds, bats, and insects from our analysis (Byers, 2011; Chapman, Drake, & Reynolds, 2011). While it is challenging to differentiate taxa with absolute certainty using weather surveillance radar, evidence from the literature suggests that measures (e.g., reflectivity) bias toward larger reflectors, and during nighttime, those tend to be migratory birds (Stepanian et al., 2016). It is also important to note that, due to the radar's limited ability to detect the lowest movements above ground level, some aerial activity may have gone undetected, particularly during the summer period, when migration is largely absent and low‐altitude nonmigratory movements may dominate. Although this may introduce some variability in flight height estimates, especially near the lower detection limit, our analysis is designed to quantify where and when aerial activity is concentrated, rather than to distinguish the behavioral context of flight (e.g., takeoff, landing, or active migration). Our focus remains on the spatial distribution of aerial density, not the behavioral mechanisms driving flight altitude.

We observed broad macroscale patterns in our measures of percent of nocturnality, peak timing, and flight heights. Higher nocturnal activity at northern latitudes may reflect a geographic trend toward lower abundance of diurnally migrating insects with increasing latitude, as hypothesized in the introduction. Tielens and Kelly (2024) showed the greatest concentration of diurnal insect density in the south‐central United States, showing approximately a 4‐fold difference across latitudinal extremes. We also observed that migration activity peaked later at northern latitudes and at more westerly sites and flight heights increased with decreasing longitude (i.e., westward). These patterns may relate to topography, particularly the mountainous terrain of the western United States, which introduces greater variability in above‐ground flight altitudes and may require migrants to fly longer to lift into the sampling space of the radar (Gauthreaux, 1991). Herein, we describe some of these specific patterns.

As early as the 1950s, moonwatchers found that autumn migration peaked between 2200 and 2300 local time (Lowery & Newman, 1966). More recently, weather surveillance radar data in the northeastern United States showed autumn peaks in the first 20%–40% of the night, which translates to 3–4 h after sunset (Farnsworth et al., 2016). Our finding of peak activity approximately 4.1 ± 0.7 (±SD) and 4.0 ± 0.8 (±SD) hours after sunset for spring and autumn, respectively, broadly corresponds to earlier findings. However, there was substantial geographic variation in timing, with 7.7% station–season (*n* = 22) combinations peaking less than 3 h after sunset and 9.4% of combinations (*n* = 27) peaking more than 5 h after sunset. Early peaks were almost exclusively tied to coastal locations (20 of 22), with peak migration activity occurring earliest surrounding the Gulf of Mexico in the spring (e.g., Figure 1B). Later peaks (>5 h) were more variably distributed, but Key West, Florida (KBYX, Figure 1D) consistently showed late peaks, as passage through these airspaces was driven by distant mainland locations (e.g., Cuba in the spring, mainland Florida in autumn).

An investigation of six NEXRAD sites in the eastern United States found that mean heights were between 400 and 600 m above ground level (agl) (Horton et al., 2016), which aligns broadly with our observation of a mean lower boundary of 355 m agl for the vertical envelope encompassing 50% of aerial activity, and a mean envelope width of 516 m. These findings contrast with radar studies in Europe and Africa, where the highest concentration of activity generally occurred below 200 m (Bradarić et al., 2024) or between 200 and 400 m (Bruderer et al., 2018). When examining differences across seasons, we found that envelopes of activity were slightly wider during the autumn by ~55 m; however, when examining coastal western US sites, all showed wider envelopes and higher envelope starting heights in the spring compared to the autumn. This seasonal difference was most dramatic at the San Joaquin Valley site (KHNX, Figure 1C), which showed an envelope width of 730 m in spring, starting at 1380 m agl, as compared to an envelope width of 560 m in autumn, starting at 332 m agl This site emphasizes the importance of examining the surrounding landscape when quantifying aerial space use. KHNX is positioned in a valley (94 m above sea level) surrounded by the Sierra Nevada mountains and Southern Coastal Ranges. This suggests that migrants differentially use the surrounding habitat across seasons, likely lifting off nearby mountainous stopover habitats in spring, and valley habitats in autumn. Stopover mapping in this region supports these seasonal differences (Horton et al., 2023).

The potential applications of our findings underscore the importance of defining the aerial space use for migratory animal conservation guidance. Flight height data provide useful information for assessing the collision risk of migratory animals with human infrastructure. Risk arises in locations where aerial animals' flight height and wind turbine rotor‐swept area overlap (Cohen et al., 2022). Such risk has been demonstrated, for example, through models that compare species‐specific flight heights with the rotor‐swept zones of offshore turbines in Europe (Borkenhagen et al., 2017; Johnston et al., 2014). Having the ability to predict the time at which the greatest proportion of migrating organisms is in the overlapping area can be useful in mitigating their risk of collisions. Moreover, by understanding the peak timing of migration activities of birds, bats, and insects, conservation actions can be taken through anticipating and mitigating potential conflicts between migratory animals and human activities with dynamic and optimized approaches (Burt, Kelly, Fox, et al., 2023; Horton et al., 2021). Targeted lights‐out guidelines that reduce the number of lights‐out nights while covering the peak migration nights during peak hours can be effective for reducing collisions and require a lower social cost of conservation (Burt, Kelly, Fox, et al., 2023).

We demonstrate radar as a powerful tool for describing biological systems in the airspace across broad extents, but also note limitations that could serve as the basis for future research. Birds have served as a model taxon for resource partitioning theory (MacArthur, 1958) and empirical studies have demonstrated this concept throughout other portions of their full annual cycle (Holm & Burger, 2002; Kent & Sherry, 2020). Yet, this concept remains understudied for birds in flight and other sources of data could shed light on taxonomic patterns in aerial space use that we describe. Notably, weather radar cannot currently provide insight on detailed taxonomic composition, overlooking the potential for intra‐ and interspecific competition, resulting niche partitioning amongst migrants in the airspace. Biologically dedicated radars, which have the ability to classify targets within guilds, have become more prevalent (Giuntini et al., 2024; Jimenez et al., 2024; Simons et al., 2025; Weisshaupt et al., 2023), and could provide more information on the relative flight heights of different functional groups. Further, extrinsic tracking data, such as activity and pressure‐based loggers, can serve as a rich source of individual flight behavior of small birds (e.g., Bäckman et al., 2017; Sjöberg et al., 2018) and can offer additional insights into patterns of space use based on age and sex (McKinnon & Love, 2018). Given key advances in nocturnal flight call monitoring (Osterhaus et al., 2025; Van Doren et al., 2023), expansions in extrinsic tracking technology (Scarpignato et al., 2023), and a growing emphasis on data integration to study migratory birds (Meehan et al., 2022), we suggest that complementing radar data with compositional information could serve as an area ripe for refining our understanding of airspace use across the full annual cycle of migratory aerofauna.

Competition has shaped species' terrestrial distributions, but has it shaped the aerial habitat use? During migration, there are clear pressures to force partitioning of limited foraging resources, increasing both intraspecific and interspecific competition. However, the factors leading to these ground‐based density‐dependent dynamics vary greatly in airspace. During active migration, favorable winds may be the dominant resource (Alerstam, 1979; Alerstam et al., 2003; Kranstauber et al., 2015; Liechti, 2006)—which may not be in short supply. With ample resources available during active flight (e.g., wind profit), airspaces may be a habitat where partitioning does not play out. Many species have been shown to comigrate (Cohen & Satterfield, 2020; DeSimone et al., 2024); however, quantifying these behaviors during active nocturnal flight can be challenging. Flight call monitoring offers species‐specific insights into comigration (Van Doren et al., 2025). While the exact function of flight calls is unknown, it is thought to aid in communication during flight—moreover, multiple species, particularly wood‐warblers, have seemingly identical flight calls (Farnsworth, 2005). Gayk and Mennill (2023) found that species with more similar calls (e.g., zeep calls) were more apt to comigrate. If airspaces are not partitioned by competition, we may predict airspace resources to be shared at the community level, with flight calls potentially acting as an interspecific communication mechanism—to this end, radar observations are well aligned to generalize this communal habitat use. Using radar, we define spatial and temporal dimensions of airspace habitat use of migratory animals, but clearly, many more elements remain to be considered to assess the suitability of such habitat for migrants (e.g., wind speed, direction, temperature, visibility, etc.). The conservation of migratory species and their habitats is crucial to maintaining the balance and health of ecosystems, both locally and globally, which are integrally connected by aerial habitats.

## AUTHOR CONTRIBUTIONS

All authors contributed to writing, editing, and engaging in discussions to frame this manuscript. Sheldon and Maji processed level‐II NEXRAD data. Horton conceptualized the study, led the analysis, and generated figures. Giuntini, Burt, and Horton led the first draft of this manuscript.

## CONFLICT OF INTEREST STATEMENT

The authors declare no conflicts of interest.

## Data Availability

Processed weather surveillance radar data are available in Sheldon et al. (2023). Summarized data, including seasonal flight envelopes and timing of peak migration (Horton, 2025c), are available in Figshare at https://doi.org/10.6084/m9.figshare.26751586.v4. The percentage of diel activity data (Horton, 2025a) is available in Figshare at https://doi.org/10.6084/m9.figshare.29661518.v3. Code (Horton, 2025b) is available in Figshare at https://doi.org/10.6084/m9.figshare.30002608.v1.
